# Complete mitochondrial genome of the freshwater monogonont rotifer *Brachionus angularis* (Rotifera, Brachionidae)

**DOI:** 10.1080/23802359.2020.1835578

**Published:** 2020-11-13

**Authors:** Min-Sub Kim, Beom-Soon Choi, Erick Ochieng Ogello, Hee-Jin Kim, Atsushi Hagiwara, Jae-Seong Lee

**Affiliations:** a Department of Biological Sciences, College of Science, Sungkyunkwan University, Suwon, South Korea; b Phyzen Genomics Institute, Seongnam, South Korea; c Department of Fisheries & Natural Resources, Maseno University, Kisumu, Kenya; d Institute of Integrated Science and Technology, Nagasaki University, Nagasaki, Japan; e Organization for Marine Science and Technology, Nagasaki University, Nagasaki, Japan

**Keywords:** Monogonont rotifer, complete mitochondrial genome, *Brachionus angularis*, Kenyan strain

## Abstract

The two complete mitochondrial genomes were sequenced from the freshwater monogonont rotifer *Brachionus angularis*. The mitochondrial genome sequences were 10,764 bp (mitochondrial DNA I) and 12,238 bp (mitochondrial DNA II) in size, respectively. The gene structure and its orientation of 12 protein-coding genes (PCGs) of complete mitochondrial genomes of *B. angularis* was identical to those shown in other marine rotifers and the freshwater rotifer *Brachionus rubens*, but was different from the freshwater rotifer *Brachionus calyciflorus*. Of 12 PCGs, one gene (*ND5*) had incomplete stop codon. Furthermore, the start codon for *CO1*, *ND4L*, *ND5*, and *CO2* was GTG, while the start codon for *ND3* and other PCGs was ATA and ATG, respectively. The base composition of 12 PCGs in *B. angularis* mitogenome showed 20.4% for A, 47.3% for T, 17.5% for C, and 14.8% for G, respectively. The mitochondrial genome A + T base composition (67.7%) of 12 PCGs was higher than G + C (32.3%), while the complete mitochondrial genome A + T base composition (66.3%) was higher than G + C (33.7%).

The freshwater rotifer *Brachionus angularis* consists of at least four subspecies (*Brachionus angularis angularis*, *Brachionus angularis bidens*, *Brachionus angularis caudatus*, and *Brachionus angularis dolabratus*) (Hu et al. 2003; Segers 2007; Hu and Xi 2008). However, to date, there is no report on complete mitochondrial genome of *B. angularis*, while several complete mitochondrial genome of other *Brachionus* rotifers have been published from *Brachionus plicatilis*, *Brachionus koreanus*, *Brachionus rotundiformis*, *Brachionus calyciflorus*, *Brachionus paranguensis*, and *Brachionus rubens* (Suga et al. 2008; Hwang et al. 2013, 2014; Kim et al. 2017; Choi et al. 2019; Choi, Kim, et al. 2020; Choi, Lee, et al. 2020). Thus, the revealing of complete mitochondrial genome of *B. angularis* would be helpful to better understand the phylogenetic relationship of *B. angularis* species complex clade. Also, *B. angularis* is considered as a model for aquaculture (Ogata et al. 2011; Ogello and Hagiwara 2015; Ogello et al. 2016), environmental biology (Ferrão-Filho et al. 2002; Wang et al. 2016), and ecology (Yang et al. 2009; Zhang et al. 2010; Yin et al. 2017) in response to environmental factors. In this study, we identified two complete mitochondrial genomes of the monogonont rotifer *B. angularis*.

The resting eggs of *B. angularis* were collected by Dr. E.O. Ogello (Kenya Marine and Fisheries Research Institute in Kenya) by netting from sediments of freshwater ponds (0°42′35.7′′S and 34°49′11.3′′E) in August 2014, then transported to the Laboratory of Aquaculture Biology, Nagasaki University, Japan for further study (Ogello et al. 2016). To identify complete mitochondrial DNA of *B. angularis*, the live samples were sent to South Korea. The type of *B. angularis* (85.6 μm in length and 75.4 μm in width) was deposited at the Ichthyological collection of the Faculty of Fisheries, Nagasaki University (FFNU) under the accession no. FFNU-Rot-0006.

We sequenced *B. angularis* from whole body genomic DNA using the nanopore platform (Oxford Nanopore Technologies, Oxford, United Kingdom). *De novo* assembly was conducted by smartdenovo (https://github.com/ruanjue/smartdenovo). For the assembled *B. angularis* 106 contigs (63,578,663 bp), Pilon version 1.23 (https://github.com/broadinstitute/ pilon/releases) and the 300 bp HiSeq2500 (Illumina, San Diego, CA) data were employed for polishing processes, and obtained one complete mitochondrial DNA sequence (mitochondrial DNA I) through manual editing process. In addition, to identify the second complete mitochondrial genome (mitochondrial DNA II), we revisited and examined the initially assembled 134,733 contigs (80,281,057 bp), generating from *de novo* assembly of 300 bp HiSeq2500 (Illumina) data with Newbler version 2.9 (http://www.454.com), based on the complete mitogenome of the freshwater rotifer *B. rubens*.

The complete mitochondrial genomes of *B. angularis* were 10,764 bp (mitochondrial DNA I; GenBank no. MT875425) and 12,238 bp (mitochondrial DNA II; GenBank no. MT875426) in size. The gene structure and its orientation of 12 PGCs of complete mitochondrial genomes of *B. angularis* were identical to those shown in other marine rotifers and the freshwater rotifer *B. rubens*, but was different from the freshwater rotifer *B. calyciflorus* that had a different combination of 12 PGCs with additional cytochrome b gene in the mitochondrial DNA I. Of 12 protein-coding genes (PCGs), one gene (*ND5*) had incomplete stop codon. Furthermore, GTG was identified as the start codon for *CO1*, *ND4L*, *ND5*, and *CO2* while ATA was the start codon for *ND3*, whereas the start codon for other PCGs was ATG. The base composition of 12 PCGs in *B. angularis* mitogenome showed 20.4% for A, 47.3% for T, 17.5% for C, and 14.8% for G, respectively. The mitochondrial genome A + T base composition (67.7%) of 12 PCGs was higher than G + C (32.3%), whereas the complete mitochondrial genome A + T base composition (66.3%) was higher than G + C (33.7%).

The placement of *B. angularis* in the genus *Brachionus* with 12 PGCs was shown in Figure 1. *B. angularis* was clustered with *B. rubens* and *B. calyciflorus* which are freshwater species, but was clearly separated from the marine species such as *B. rotundiformis*, *B. koreanus*, and *B. paranguensis*, possibly suggesting relationship between the differences in their natural habitat and mitogenome.

**Figure 1.**
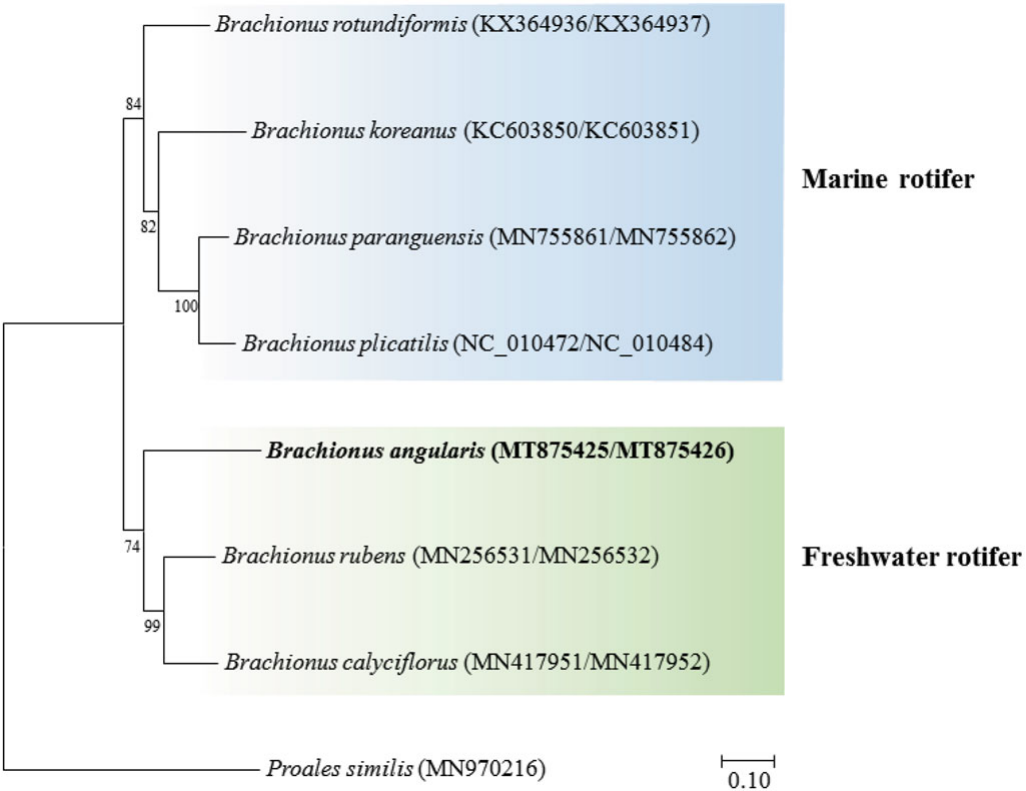
Phylogenetic analyses based on mitochondrial DNA of *Brachionus angularis* with seven congeners. The amino acid sequences of 12 mitochondrial DNA genes were aligned by ClustalW. Maximum likelihood analysis was performed by Mega software (version 10.0.1) with Gamma + LG + I model. The rapid bootstrap analysis was conducted with 1000 replications with 48 threads running in parallel. The rotifer *Proales similis* (class Monogononta) served as outgroup. −Ln = 28545.407996.

## Data Availability

The data that support the findings of this study are openly available in GenBank of NCBI at https://www.ncbi.nlm.nih.gov, reference numbers MT875425 and MT875426.
